# Staring Imaging Attitude Tracking Control Laws for Video Satellites Based on Image Information by Hyperbolic Tangent Fuzzy Sliding Mode Control

**DOI:** 10.1155/2022/8289934

**Published:** 2022-08-10

**Authors:** Wenjing Pei

**Affiliations:** The Seventh Research Division and the Center for Information and Control, School of Automation Science and Electrical Engineering, Beihang University (BUAA), Beijing 100191, China

## Abstract

This paper studies the staring imaging attitude tracking and control for satellite videos based on image information. An improved temporal-spatial context learning algorithm is employed to extract the image information. Based on this, a hyperbolic tangent fuzzy sliding mode control law is proposed to achieve the attitude tracking and control. Furthermore, the hyperbolic tangent function and fuzzy logic system are introduced into the sliding mode controller. In the experiments, the improved temporal-spatial context learning algorithm is applied for the image information of the space target video sequence captured by Jilin-1 in orbit, where the image information is used as the input of the control loop. Moreover, the proposed method is realized through simulation. Besides, the image change caused by attitude adjustment is achieved successfully, and the target imaging can be located in the center of the image plane to realize the gaze tracking control of the space target effectively.

## 1. Introduction

With the rapid development of remote sensing technology, video satellites have attracted much attention due to their continuous observation ability [1–8]. As a new type of Earth observation satellite, video satellites can employ the payload on the satellite platform to be pushed scan imaging with the help of the orbital motion of the satellite. Note that video satellites are to adjust the attitude real-timely and to obtain the dynamic information of the target area continuously in the process of staring imaging, so the optical axis of the optical load points at the target at all the time. In Ref. [9], Liu et al. introduce the gaze imaging technique, where the process generally refers to the staring attitude control. In addition, video satellites can use agility and attitude control technology to realize continuous imaging of ground targets. Due to this reason, compared with the traditional Earth observation satellites, video satellites have widely applied in many fields, such as vehicle real-time monitoring [10, 11], rapid response to natural disaster emergency [12], and major engineering monitoring [13].

**Figure 1:**
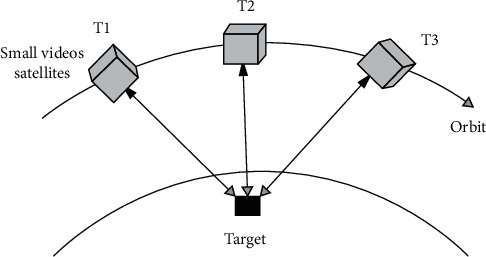
Schematic diagram of ground gazing attitude control of the video satellite.

Up to now, there are mainly two types of staring imaging satellites in orbit, including the satellites in the geostationary orbit and video satellites in the low orbit [14–16].  Figure 1 shows the schematic diagram of the ground gazing attitude control of video satellites. Furthermore, the attitude control system in the video satellites can adjust the attitude in real-time so that the optical axis of the optical sensor always points to the ground target area for continuous photography. Staring imaging is the main working mode for video satellites [17, 18]. In essence, although the staring imaging control problem is a dynamic attitude tracking problem, it is difficult to ensure the high stability of the optical axis of the satellite optical sensor to the observed object.

In the last decades, the staring image attitude control for video satellites can be regarded as a spacecraft attitude tracking and control problem [19–21]. Much research work has been done on the attitude tracking control for the satellites. Meanwhile, some controllers have been employed in satellites attitude control, such as sliding mode controller, robust controller, and intelligent controller [19–34].

So far, compared with attitude tracking and control for spacecraft and satellites, several satellite staring attitude control methods have been introduced to achieve real-time tracking [24–32]. For instance, Lian et al. [27] investigated the small satellite attitude problems for staring operation. Liang et al. [28] designed the fuzzy logic control law for the staring imaging satellite attitude problem in LEO, which has a quick response and excellent robustness. In Ref. [29], Chen et al. present a quaternion-based PID feedback tracking controller with the gyroscopic term cancellation, where some desired target can be tracked on the Earth. Chen et al. [30] investigated a staring imaging attitude controller based on double-gimbaled control moment gyroscope (DGCMG), which is simple and effective for agile small satellites. In Ref. [31], Li and Liang proposed a robust finite-time controller aiming at satellite attitude maneuvers to demonstrate the robustness of some typical perturbations such as disturbance torque, model uncertainty, and actuator error. Li et al. [32] implemented the neural network controller for staring imaging, where the real-time performance can be achieved.

However, the above staring attitude controller does not consider the image information directly. Moreover, the image information is separated from the attitude tracking controller. Besides, we note that in the satellite staring mode, the optical axis of the camera should point fixedly to the target for a long time. Meanwhile, both the video satellites and the object may be moving in the inertial coordinate system. In this way, the relative velocity and position may be changing over time.

In essence, visual information is introduced into the closed-loop control, which is commonly known as visual servo, and is first applied in the field of robots [35–38]. Recently, robot visual servo has achieved numerous results in both theory and practical application. We note that robot visual servo is generally divided into two structures: the position-based visual servo and the image-based visual servo. The position-based servo should calibrate the internal parameters of the camera to determine the relative attitude between the target and the camera coordinate system, which may increase the amount of calculation of the system. On the contrary, the image-based visual servo directly uses the visual feature error of the target in the phase plane and takes the controlled object and the visual system as a whole.

Due to the above reasons and advantages, visual information was initially introduced into the control closed loop of satellites and spacecraft. Therefore, an improved temporal-spatial context learning (ISTC) algorithm is employed to extract the image information in this paper. Based on this, a hyperbolic tangent fuzzy sliding mode control law (HTFSMC) for small video satellites is designed to achieve the attitude tracking and control. In special, the related coordinates are defined for attitude transformation. Subsequently, the sliding mode tracking controller is presented based on the image information from satellite videos. Furthermore, the hyperbolic tangent function and the fuzzy logic system are employed in the sliding mode controller.

In summary, the contributions of this paper are threefold.

1. In this paper, an ISTC algorithm is employed to obtain the image information. Hence, the visual information can be employed effectively in the visual tracking control based on spatial moving images, instead of cumbersome, complex camera internal parameter calibration, and accurate information of target and camera motion.
2. Based on the image information, this paper proposed the HTFSMC, where the hyperbolic tangent function and fuzzy logic system are introduced into the sliding mode controller.
3. In the experiments, the image information of the space target video sequence captured by Jilin-1 in orbit is used as the input of the controller. The control part is realized through simulation. Besides, the image change caused by attitude adjustment is achieved successfully, and the target imaging can be located in the center of the image plane to realize the gaze tracking control of the space target effectively.

The rest of this paper is arranged as follows. In Section 2, the staring imaging attitude dynamics model is described in detail. In what follows of this section, the sliding mode controller and fuzzy sliding controller are presented for staring imaging attitude tracking of small satellite videos. In Section 3, the experiment results and some discussion are introduced. Finally, Section 4 concludes this article.

## 2. Materials and Methods

**Figure 2:**
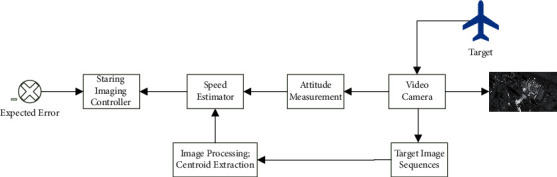
Structure frame of staring imaging attitude tracking control system based on image information.

In this paper, in order to extract the image information, the ISTC is employed for moving object tracking. Based on this, an attitude controller based on image information feedback is designed to realize the gaze tracking control of moving targets. The structure diagram is shown in Figure 2. In a nutshell, this method is to conform the centroid coordinates and to calculate the position deviation from the image center. Therefore, the cumbersome process of camera calibration and relative pose estimation can be avoided so that the computation cost is reduced.

### 2.1. An Improved Spatio-Temporal Context Learning Algorithm

For moving target video tracking, the local context is the background of the target and an certain area nearby. In fact, there is a strong space-time relationship in the local scene around the target between consecutive frames. According to the space-time relationship between the target and its surrounding area, the spatio-temporal context (STC) learning algorithm is constructed a spatio-temporal context model for the target and the nearby area based on the gray features of the image. Moreover, the confidence map of the target is to be calculated, and the maximum-likelihood probability in the confidence map can be found as the estimated target position. Therefore, the ISTC algorithm is described in detail.

The confidence map of the target position can be set as *x*^*∗*^. The luminance feature set of *x*^*∗*^ is defined as

(1) $$\begin{array}{c} X^{c}=\left\{c\left(z\right)=\left(I\left(z\right),z\right)|z\in \Omega_{c}\left(x^{*}\right)\right\}, \end{array}$$

where *c*(*z*) and *I*(*z*) are the luminance feature and the image intensity at position *z*, respectively. Ω_*c*_(*x*^*∗*^) denotes the context area of position *x*^*∗*^. In the following, *c*(*x*) can be used to calculate the confidence map as follows:

(2) $$\begin{array}{c} c\left(x\right)=P\left(x|o\right),\\ =\sum_{c\left(z\right)\in X^{c}}P\left(x,c\left(z\right)|o\right),\\ =\sum_{c\left(z\right)\in X^{c}}P\left(x|c\left(z\right),o\right)P\left(c\left(z\right)|o\right), \end{array}$$

where *P*(*x|c*(*z*), *o*) is the conditional probability, which can represent the spatial relationship between the target position and its the context information. In addition, *P*(*c*(*z*)*|o*) is the prior probability, which can model the appearance of the local context. Furthermore, *P*(*x|c*(*z*), *o*) can be defined as

(3) $$\begin{array}{c} P\left(x|c\left(z\right),o\right)=h^{sc}\left(x-z\right), \end{array}$$

where *h*^*sc*^(*x* − *z*) is the relative distance and direction function between the target position *x* and its local context information. Subsequently, *P*(*c*(*z*)*|o*) can be also defined as

(4) $$\begin{array}{c} P\left(c\left(z\right)|o\right)=I\left(z\right)\omega_{\sigma }\left(z-x^{*}\right)\\ =I\left(z\right)ae^{-\left(\left|z\right|/\sigma^{2}\right)}, \end{array}$$

where *ω*_*σ*_(*z* − *x*^*∗*^) is a weight function, *a* is a normalized constant, which makes *P*(*c*(*z*)*|o*) range from 0 to 1 in (4), and *σ* is a scale parameter. Hence, the confidence map *c*(*x*) in (2) can be rewritten as

(5) $$\begin{array}{c} c\left(x\right)=P\left(x|o\right),\\ =be{\left|\frac{x-x^{*}}{a}\right|}^{\beta },\\ =\sum_{z\in \Omega_{c}\left(x^{*}\right)}h^{sc}\left(x-z\right)I\left(z\right)\omega_{\sigma }\left(z-x^{*}\right),\\ =h^{sc}\left(x\right)⊗\left(I\left(x\right)\omega_{\sigma }\left(x-x^{*}\right)\right), \end{array}$$

where ⊗ is the convolution operator and *β* is an important shape parameter. Fast Fourier transform is utilized simultaneously on both sides of the equation for (5). Therefore, (5) can be updated as

(6) $$\begin{array}{c} ℱ\left(be{\left|\frac{x-x^{*}}{a}\right|}^{\beta }\right)=ℱ\left(h^{sc}\left(x\right)\right)⊙ℱ\left(I\left(x\right)\omega_{\sigma }\left(x-x^{*}\right)\right), \end{array}$$

where ℱ is the fast Fourier transform (FFT) and ⊙ denotes the element-wise product. Subsequently, *h*^*sc*^(*x*) can be obtained as

(7) $$\begin{array}{c} h^{sc}\left(x\right)={ℱ}^{-1}\left(\frac{ℱ\left(be{\left|x-x^{*}/a\right|}^{\beta }\right)}{ℱ\left(I\left(x\right)\omega_{\sigma }\left(x-x^{*}\right)\right)}\right), \end{array}$$

where ℱ^−1^ is the inverse FFT. In this way, based on (7), the spatio-temporal context model can be derived as

(8) $$\begin{array}{c} H_{k-1}^{sc}=\left(1-\rho \right)H_{k}^{sc}+\rho h_{k}^{sc}, \end{array}$$

where *h*_*t*_^*sc*^ is the spatio-temporal context model at the *k*-th frame in (7). Hence, *h*_*t*+1_^*sc*^ can be also obtained by (7). Thus, the confidence map at the (*k*+1)-th frame is expressed as

(9) $$\begin{array}{c} c_{t+1}\left(x\right)={ℱ}^{-1}\left(ℱ\left(H_{k+1}^{sc}\left(x\right)⊙ℱ\left(I_{k+1}\left(x\right)\omega_{\sigma }\left(x-x_{k}^{*}\right)\right)\right)\right). \end{array}$$

The confidence map is maximized, so the location of the target can be obtained as

(10) $$\begin{array}{c} x_{k+1}^{*}=\arg\underset{X\in \Omega_{c}\left(x_{k}^{*}\right)}{\max}c_{t+1}\left(x\right). \end{array}$$

Since the target attitude may be changed in the process of target movement, the size of the target may be also changed. Besides, the background information may be different in each frame. Therefore, the scale update strategy can be employed for the target, which is given as

(11a) $$\begin{array}{c} s_{t}^{\prime }=\sqrt{\frac{c_{t}\left(x_{t}^{*}\right)}{c_{t-1}\left(x_{t-1}^{*}\right)}}, \end{array}$$

(11b) $$\begin{array}{c} \bar{s_{t}}=\frac{1}{n}\sum_{i=1}^{n}s_{t-1}^{\prime }, \end{array}$$

(11c) $$\begin{array}{c} s_{t+1}=\left(1-\lambda \right)s_{t}+\lambda \bar{s_{t}}, \end{array}$$

(11d) $$\begin{array}{c} \sigma_{t+1}=s_{t}\sigma_{t}. \end{array}$$

However, in Equation (11a), the denominator may be close to zero so that the results of moving object tracking may occasionally lead to overfitting. Due to this reason, an improved scale update strategy is introduced to avoid an abrupt change based on a penalty term *p*(*s*):

(12) $$\begin{array}{c} p\left(s\right)=\left\{\begin{array}{c} \varsigma ,\;\log_{2}\left(s\right)<\varsigma ,\\ -\;\log_{2}\left(s\right),\left|\log_{2}\left(s\right)\right|\leq \varsigma \\ -\varsigma ,\;\log_{2}\left(s\right)>\varsigma , \end{array}\right., \end{array}$$

where *ς* is a constant. In this way, the updated scale can be rewritten as

(13) $$\begin{array}{c} s_{t}^{\prime }=s_{t+1}+p\left(s_{t+1}\right). \end{array}$$

### 2.2. Staring Imaging Attitude Dynamics Model

#### 2.2.1. The Definitions of the Related Coordinate Systems

**Figure 3:**
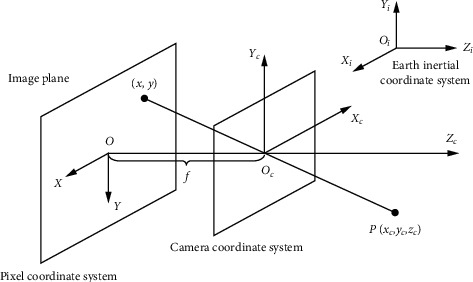
Schematic diagram of ground gazing attitude control of video small satellite.

In this paper, some related coordinate systems are shown in Figure 3. The inertial coordinate system of Earth is defined as *O*_*i*_ − *X*_*i*_*Y*_*i*_*Z*_*i*_, where the coordinate origin *O*_*i*_ is located at the center of mass of the Earth. The *X*_*i*_-axis is on the equatorial plane, which is pointing at the vernal equinox of the time. The direction of *Z*_*i*_-axis is the consistent with the Earth rotation axis. The *Y*_*i*_-axis is in the equatorial plane and meets the right-hand orthogonal reference. The satellite body coordinate system is defined as *O*_*b*_ − *X*_*b*_*Y*_*b*_*Z*_*b*_, where the center of mass of the satellite is the origin of the coordinate system *O*_*b*_. The image coordinate system is *O* − *X*_*p*_*Y*_*p*_. The camera coordinate system is *O*_*c*_ − *X*_*c*_*Y*_*c*_*Z*_*c*_. The image pixel coordinate system is *I* − *xy*.

#### 2.2.2. The Attitude Solution Based on Satellite Images

**Figure 4:**
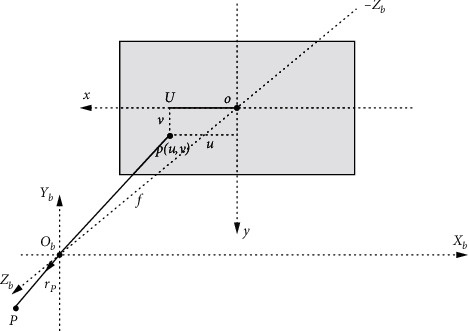
The diagram of target deviation on image plane.

In this paper, we assume that the camera coordinate system *O*_*c*_ − *X*_*c*_*Y*_*c*_*Z*_*c*_ coincides with the satellite coordinate system *O*_*b*_ − *X*_*b*_*Y*_*b*_*Z*_*b*_. The unit vector in the *O*_*b*_*Z*_*b*_ direction is *r*=(0,0,1)^*T*^. As shown in Figure 4, the coordinates of target *P* is set as (*u*, *v*) in the pixel coordinate system *I* − *xy*. In the satellite coordinate system *O*_*b*_ − *X*_*b*_*Y*_*b*_*Z*_*b*_, the target line of sight direction *r*_*P*_ can be described as

(14) $$\begin{array}{c} r_{P}=\frac{1}{\sqrt{l^{2}m^{2}+l^{2}n^{2}+f^{2}}}\left(\begin{array}{c} lu\\ lv\\ f \end{array}\right)\\ =\left(\begin{array}{c} x_{P}\\ y_{P}\\ z_{P} \end{array}\right), \end{array}$$

where (*x*_*P*_, *y*_*P*_, *z*_*P*_) is the coordinate of the target in the satellite coordinate system *O*_*b*_ − *X*_*b*_*Y*_*b*_*Z*_*b*_. The focal length of the spaceborne camera is *f* and the pixel size is *l*.

The purpose is to ensure the target can image in the center of the image. Hence, it is necessary to coincide with *r* and *r*_*P*_. In the process of staring tracking, the images are required to remain stable and no rotation, which is convenient for image observation and analysis. Staring tracking imaging is the process of controlling *r* to track *r*_*P*_.

In the satellite coordinate system *O*_*b*_ − *X*_*b*_*Y*_*b*_*Z*_*b*_, the following assumptions are given as

(15a) $$\begin{array}{c} O_{b}=\left(-lu,0,-f\right), \end{array}$$

(15b) $$\begin{array}{c} r_{1}=-\frac{O_{b}U}{O_{b}U}, \end{array}$$

First, we rotate around the *O*_*b*_*Y*_*b*_ axis so that *r* is coincided with *r*_1_. The rotation angle *θ* is expressed as

(16) $$\begin{array}{c} \theta =\arctan\frac{lu}{f}. \end{array}$$

Then, we rotate around the *O*_*b*_*X*_*b*_ axis so that *r* is coincided with *r*_*P*_. The rotation angle *φ* is demonstrated as

(17) $$\begin{array}{c} \varphi =\arctan\frac{-lv}{\sqrt{f^{2}+u^{2}}}. \end{array}$$

The attitude quaternions *q* are defined as

(18a) $$\begin{array}{c} q=\left(\begin{array}{c} q_{0}\\ \bar{q} \end{array}\right), \end{array}$$

(18b) $$\begin{array}{c} \bar{q}={\left(q_{1},q_{2},q_{3}\right)}^{T}, \end{array}$$

(18c) $$\begin{array}{c} q^{T}q=q_{0}^{2}+q_{1}^{2}+q_{2}^{2}+q_{3}^{2}, \end{array}$$

where $\bar{q}$ is the vector part, and *q*_0_ is the scalar part. The quaternion is obtained as the equation (19) through rotating *θ* around (0,1,0)^*T*^.

(19) $$\begin{array}{c} q_{1}={\left(\cos\frac{\theta }{2},0,\;\sin\frac{\theta }{2},0\right)}^{T}. \end{array}$$

Through rotating *φ* around (0,1,0)^*T*^, the quaternion is obtained as

(20a) $$\begin{array}{c} q_{2}={\left(\cos\frac{\varphi }{2},\;\sin\frac{\varphi }{2},0,0\right)}^{T}, \end{array}$$

(20b) $$\begin{array}{c} q_{2}⊗q_{1}=\left(\begin{array}{c} q_{0}^{\left(1\right)}q_{0}^{\left(2\right)}-{\bar{q}}^{\left(1\right)}{\bar{q}}^{\left(2\right)}\\ q_{0}^{\left(1\right)}{\bar{q}}^{\left(2\right)}+q_{0}^{\left(2\right)}{\bar{q}}^{\left(1\right)}-{\bar{q}}^{\left(2\right)}\times {\bar{q}}^{\left(1\right)} \end{array}\right). \end{array}$$

where ⊗ is the rotation multiplication operator of quaternions. Thereby, the expected attitude error quaternion *q*_*e*_ is expressed as

(21) $$\begin{array}{c} q_{e}={\left(q_{2}⊗q_{1}\right)}^{-1},\\ ={\left(\begin{array}{c} q_{0}^{\left(1\right)}q_{0}^{\left(2\right)}-{\bar{q}}^{\left(1\right)}{\bar{q}}^{\left(2\right)}\\ q_{0}^{\left(1\right)}{\bar{q}}^{\left(2\right)}+q_{0}^{\left(2\right)}{\bar{q}}^{\left(1\right)}-{\bar{q}}^{\left(2\right)}\times {\bar{q}}^{\left(1\right)} \end{array}\right)}^{-1},\\ =\left(\begin{array}{c} \begin{array}{c} \cos\frac{\varphi }{2}\cos\frac{\theta }{2}\\ -\sin\frac{\varphi }{2}\cos\frac{\theta }{2} \end{array}\\ \begin{array}{c} -\cos\frac{\varphi }{2}\sin\frac{\theta }{2}\\ -\sin\frac{\varphi }{2}\sin\frac{\theta }{2} \end{array} \end{array}\right). \end{array}$$

Therefore, the expected attitude quaternion *q*_*t*_ is relative to the Earth inertial system can be expressed as

(22) $$\begin{array}{c} q_{t}=q_{e}^{-1}⊗q_{b}, \end{array}$$

where *q*_*b*_ is attitude quaternion of satellite body coordinate system relative to the Earth inertial system.

The attitude kinematics equation is shown as

(23a) $$\begin{array}{c} {\dot{q}}_{t}=\frac{1}{2}E\left(q_{t}\right)\omega_{t}, \end{array}$$

(23b) $$\begin{array}{c} E\left(q_{t}\right)=\left(\begin{array}{c} -{\bar{q}}^{T}\\ {\bar{q}}^{\times }+q_{0}I_{3\times 3} \end{array}\right), \end{array}$$

(23c) $$\begin{array}{c} {\bar{q}}^{\times }=\left(\begin{array}{ccc} 0 & -q_{3} & q_{2}\\ q_{3} & 0 & q_{1}\\ q_{2} & q_{1} & 0 \end{array}\right), \end{array}$$

where ${\bar{q}}^{\times }$ is an antisymmetric matrix, and *I*_3×3_ is an identity matrix. Then, the following equation is verified as

(24) $$\begin{array}{c} E{\left(q_{t}\right)}^{T}E\left(q_{t}\right)=I_{3\times 3}. \end{array}$$

Using the equation (23a), the expected angular velocity *ω*_*t*_ is inversely solved as

(25a) $$\begin{array}{c} \omega_{t}=2\Xi^{T}\left(q_{t}\right){\dot{q}}_{t}. \end{array}$$

(25b) $$\begin{array}{c} \Xi \left(q_{t}\right)=\left(\begin{array}{c} -{\bar{q}}^{T}\\ q_{0}I_{3\times 3}-{\bar{q}}^{\times } \end{array}\right), \end{array}$$

The expected attitude error angular velocity is given as

(26) $$\begin{array}{c} \omega_{e}=\omega_{b}-A\left(q_{e}\right)\omega_{t}. \end{array}$$

If $q=\left(\begin{array}{c} q_{0}\\ \bar{q} \end{array}\right)$ is any quaternion, we can obtain

(27) $$\begin{array}{c} A\left(q_{e}\right)=\left(q_{0}^{2}-{\bar{q}}^{2}\right)I_{3\times 3}-2q_{0}{\bar{q}}^{\times }+2\bar{q}{\bar{q}}^{T}, \end{array}$$

where *A*(*q*_*e*_) is the attitude matrix determined by *q*_*e*_.

### 2.3. Sliding Mode Controller

In this paper, based on the actuator of three orthogonal mounted reaction flywheel, the satellite attitude dynamics equation is given as

(28) $$\begin{array}{c} J{\dot{\omega }}_{b}=-\omega_{b}\times \left(J\omega_{b}+h\right)+u+d, \end{array}$$

where *J* is the inertia moment of the satellite, *ω*_*b*_ is angular velocity of satellite body coordinate system, *h* is the angular momentum of the flywheel, *u* is control torque, and *d* is external disturbance torque.

In this paper, the sliding mode function is designed as

(29a) $$\begin{array}{c} \zeta =\omega_{e}+K{\bar{q}}_{e}m, \end{array}$$

(29b) $$\begin{array}{c} {\dot{\bar{q}}}_{e}=\frac{1}{2}F\left(q_{e}\right)\omega_{e}, \end{array}$$

(29c) $$\begin{array}{c} F\left(q\right)={\bar{q}}^{\times }+q_{0}I_{3\times 3}, \end{array}$$

(29d) $$\begin{array}{c} \omega_{e}=2\Xi^{T}\left(q_{e}\right)q_{e}, \end{array}$$

(29e) $$\begin{array}{c} {\dot{\omega }}_{b}={\dot{\omega }}_{e}+A\left(q_{e}\right){\dot{\omega }}_{t}-\left[\omega_{e}^{\times }\right]A\left(q_{e}\right)\omega_{t}, \end{array}$$

where *ζ*=[*ζ*_1_, *ζ*_2_, *ζ*_3_]^*T*^, *K*=diag(*k*_*i*_), *i*=1,2,3. If *ζ*⟶0, the angular velocity of the system and the state of the angular velocity can be tracked.

The approach function method is used to obtain the sliding mode control law.

The exponential reaching law is used as

(30) $$\begin{array}{c} \dot{\zeta }=-l\zeta -\varepsilon \mathrm{sgn}\left(\zeta \right), \end{array}$$

where sgn(*s*)=[sgn(*ζ*_1_), sgn(*ζ*_2_), sgn(*ζ*_3_)], *l*=diag(*l*_*i*_), and *ε*=diag(*ε*_*i*_), *i*=1,2,3. The control quantity *u* is obtained as

(31) $$\begin{array}{c} u=JA\left(q_{e}\right){\dot{\omega }}_{t}+\omega_{b}\times \left(J\omega_{b}+h\right)+J\left[\omega_{e}^{\times }\right]A\left(q_{e}\right)\omega_{t}-\frac{1}{2}JKF\left(q_{e}\right)\omega_{e}-K_{1}\zeta -D_{1}P\left(\zeta \right), \end{array}$$

where *K*_1_ is controller parameters. We notice that the chattering of sliding mode controller is mainly caused by sgn(*ζ*) and *d*. Let *P*(*ζ*)=sgn(*ζ*). In order to reduce chattering, *P*(*ζ*) is rewritten as

(32a) $$\begin{array}{c} \hat{P}\left(s\right)=\left[\tanh\left(\frac{\zeta_{1}}{\varepsilon }\right),\;\tanh\left(\frac{\zeta_{2}}{\varepsilon }\right),\;\tanh\left(\frac{\zeta_{3}}{\varepsilon }\right)\right], \end{array}$$

(32b) $$\begin{array}{c} \tan\left(\frac{x}{\varepsilon }\right)=\frac{e^{x/\varepsilon }-e^{-\left(x/\varepsilon \right)}}{e^{x/\varepsilon }+e^{-\left(x/\varepsilon \right)}}, \end{array}$$

where the inflection point of hyperbolic tangent function is determined through the value of *ε*(*ε* > 0).

### 2.4. Stability Analysis

In order to ensure that the state of the system move from any initial point to *s*=0 in a finite time based on the designed sliding mode controller, the following assumptive conditions are given:

1. Supposed *d* is bounded, *d*_max_ ≥ *D*_2_ is boundary of *d*.
2. Supposed $\underset{i}{\min}\left\{D_{1i}\right\}>d$.

The stability of the system is proved as follows. Lyapunov function is constructed as

(33) $$\begin{array}{c} V=\frac{1}{2}\zeta^{T}J\zeta . \end{array}$$

The derivative of the (33) is represented as

(34) $$\begin{array}{c} \dot{V}=\zeta^{T}J\dot{\zeta }\\ =\zeta^{T}\left[d-K_{1}\zeta -D_{1}P\left(\zeta \right)\right]<0, \end{array}$$

where $\dot{V}=0$ if and only if *ζ*=0. $\dot{V}$ is a seminegative definite function. Therefore, the system is convergent by using sliding mode control.

### 2.5. Fuzzy Logic System

**Figure 5:**
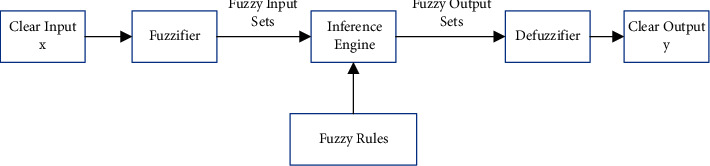
The diagram of Fuzzy logic system.

Fuzzy logic system (FLS) is consisted of fuzzy rule base, fuzzy rule base, fuzzy inference engine, and defuzzifier, as shown Figure 5. In this paper, we assume that *x*_1_ ∈ *X*_1_, *x*_2_ ∈ *X*_2_,  …,  *x*_*p*_ ∈ *X*_*p*_, and *y* ∈ *Y* are *p* input and an output, respectively. Furthermore, the fuzzy rule base is composed of *k* rules, expressed as

(35) $$\begin{array}{c} R^{\left(l\right)}:\text{IF }x_{1}\text{ is }F_{1}^{l},\ldots ,x_{p}\text{ is }F_{P}^{l},\;\text{THEN }y\text{ is }G^{l}, \end{array}$$

where *l*=1,2,…, *k*. Thereby, FLS can be employed to simplify fuzzy rules as mapping from fuzzy input sets *F*_1_^*l*^ × ⋯*F*_*p*_^*l*^ to the fuzzy output set *Y*, denoted by *F*_1_^*l*^ × ⋯*F*_*p*_^*l*^=*A*^*l*^. In this way, (35) can be rewritten as

(36) $$\begin{array}{c} R^{\left(l\right)}:F_{1}^{l}\times \cdots F_{p}^{l}⟶G^{l}=A^{l}⟶G^{l},\;l=1,2,\ldots ,k. \end{array}$$

The membership function *μ*_*R*^(*l*)^_(*x*, *y*) can be utilized to describe *R*^(*l*)^ as

(37) $$\begin{array}{c} \mu_{R^{\left(l\right)}}\left(x,y\right)=\mu_{A^{\left(l\right)}⟶G^{l}}\left(x,y\right), \end{array}$$

where *x*=(*x*_1_, *x*_2_,…,*x*_*p*_)^*T*^. Hence, (37) can be rewritten as

(38) $$\begin{array}{c} \mu_{R^{\left(l\right)}}\left(x,y\right)=\mu_{R^{\left(l\right)}}\left(x_{1},x_{2},\ldots ,x_{p},y\right),\\ =\mu_{A^{\left(l\right)}⟶G^{l}}\left(x_{1},x_{2},\ldots ,x_{p},y\right),\\ =\mu_{F_{1}^{l}}\left(x^{1}\right)⋆\mu_{F_{2}^{l}}\left(x^{2}\right)⋆\cdots ⋆\mu_{F_{p}^{l}}\left(x^{p}\right)⋆\mu_{G^{l}}\left(y\right),\\ =\left[T_{i=1}^{p}\mu_{F_{i}^{l}}\left(x^{i}\right)\right]⋆\mu_{G^{l}}\left(y\right), \end{array}$$

where ⋆ represents that multiple antecedents are juxtaposed and connected with *t* norm.

*A*_*x*_ is a fuzzy set where *p* input of *R*^*l*^ is given, and the membership function *μ*_*A*^*x*^_(*x*) is defined as

(39) $$\begin{array}{c} \mu_{A^{x}}\left(x\right)=\mu_{X_{1}}\left(x_{1}\right)\mu_{X_{2}}\left(x_{2}\right)\cdots \mu_{X_{p}}\left(x_{p}\right)=T_{i=1}^{p}\mu_{X_{i}}\left(x_{i}\right). \end{array}$$

According to each fuzzy rule, a fuzzy set *B*^*l*^ about the set *Y* is given as

(40) $$\begin{array}{c} B^{l}=A_{x}\circ R^{l}. \end{array}$$

Meanwhile, based on commutativity of *t* norm, we can obtain the membership function *μ*_*B*^*l*^_(*y*) in (41)

(41) $$\begin{array}{c} \mu_{B^{l}}\left(y\right)=\mu_{A_{x}\circ R^{l}}\left(y\right),\\ =\underset{x\in X}{\sup}\left[\mu_{A^{x}}\left(x\right)⋆\mu_{A^{l}⟶G^{l}}\left(x,y\right)\right],\\ =\underset{x\in X}{\text{sup}}\left\{T_{i=1}^{p}\mu_{X_{i}}\left(x_{i}\right)⋆\left[T_{i=1}^{p}\mu_{F_{i}^{l}}\left(x^{i}\right)\right]⋆\mu_{G^{l}}\left(y\right)\right\},\\ =\underset{x\in X}{\text{sup}}\left\{\left[\mu_{X_{i}}\left(x_{i}\right)⋆\mu_{F_{i}^{l}}\left(x^{i}\right)\right]⋆\mu_{G^{l}}\left(y\right)\right\},\\ =\mu_{G^{l}}\left(y\right)⋆\left\{\left[\underset{x_{1}\in X_{1}}{\text{sup}}\mu_{X_{1}}\left(x_{1}\right)⋆\mu_{F_{1}^{l}}\left(x^{1}\right)\right]⋆\left[\underset{x_{2}\in X_{2}}{\text{sup}}\mu_{X_{2}}\left(x_{2}\right)⋆\mu_{F_{2}^{l}}\left(x^{2}\right)\right]⋆\cdots ⋆\left[\underset{x_{p}\in X_{p}}{\text{sup}}\mu_{X_{p}}\left(x_{p}\right)⋆\mu_{F_{p}^{l}}\left(x^{p}\right)\right]\right\},\;y\in Y. \end{array}$$

Singleton fuzzifier is employed into (41) and (41), which can be rewritten as

(42) $$\begin{array}{c} \mu_{B^{l}}\left(y\right)==\mu_{G^{l}}\left(y\right)⋆\left[\mu_{F_{1}^{l}}\left(x_{1}^{\prime }\right)⋆\mu_{F_{2}^{l}}\left(x_{2}^{\prime }\right)⋆\cdots ⋆\mu_{F_{p}^{l}}\left(x_{p}^{\prime }\right)\right],y\in Y. \end{array}$$

Due to centroid defuzzifier, FLS output can be expressed as

(43a) $$\begin{array}{c} y_{c}\left(x\right)=\frac{\sum_{i=1}^{N}y_{i}\mu_{B}\left(y_{i}\right)}{\sum_{i=1}^{N}\mu_{B}\left(y_{i}\right)}, \end{array}$$

(43b) $$\begin{array}{c} B=\overset{M}{\underset{l=1}{\cup }}B^{l}, \end{array}$$

where *B* is output fuzzy set, and *y*_*c*_(*x*) is the clear output.

### 2.6. Fuzzy Sliding Mode Controller

The input and output fuzzy sets of the system are defined as

(44a) $$\begin{array}{c} \zeta \dot{\zeta }=\left\{\text{NB NM NS Z PS PM PB}\right\}, \end{array}$$

(44b) $$\begin{array}{c} \Delta D_{1}=\left\{\text{NB NM NS Z PS PM PB}\right\}, \end{array}$$

where $\zeta \dot{\zeta }$ is the input, and the variation of *D*_1_ is represented Δ*D*_1_ as the output. In Equations (44a) and (44b), NB, NM, NS, Z, PS, PM, PB are negative large, negative middle, negative small, zero, positive small, positive middle, and positive large, respectively. Therefore, the following seven rules are designed as

- R1 : If $\zeta \dot{\zeta }$ is *PB*, THEN Δ*D*_1_ is *PB*.
- R2 : If $\zeta \dot{\zeta }$ is *PM*, THEN Δ*D*_1_ is *PM*.
- R3 : If $\zeta \dot{\zeta }$ is *PS*, THEN Δ*D*_1_ is *PS*.
- R4 : If $\zeta \dot{\zeta }$ is *Z*, THEN Δ*D*_1_ is *Z*.
- R5 : If $\zeta \dot{\zeta }$ is *NS*, THEN Δ*D*_1_ is *NS*.
- R6 : If $\zeta \dot{\zeta }$ is *NM*, THEN Δ*D*_1_ is *NM*.
- R7 : If $\zeta \dot{\zeta }$ is *NB*, THEN Δ*D*_1_ is *NB*.

**Figure 6:**
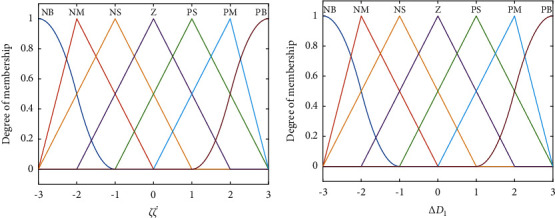
The input/output membership function of fuzzy control system: (a) membership function of fuzzy input; (b) membership function of fuzzy output.

Besides, Figure 6 shows the input/output membership function of fuzzy control system. According to the value of $\zeta \dot{\zeta }$, the centroid defuzzifier is employed and the value of Δ*D*_1_ can be obtained as $\Delta {\hat{D}}_{1}$. Meanwhile, *D*_1_ can be rewritten as

(45) $$\begin{array}{c} {\hat{D}}_{1}=D_{1}+\Delta {\hat{D}}_{1}. \end{array}$$

Hence, the fuzzy sliding mode controller can be designed as

(46) $$\begin{array}{c} u=JA\left(q_{e}\right){\dot{\omega }}_{t}+\omega_{b}\times \left(J\omega_{b}+h\right)+J\left[\omega_{e}^{\times }\right]A\left(q_{e}\right)\omega_{t}-\frac{1}{2}JKF\left(q_{e}\right)\omega_{e}-K_{1}\zeta -{\hat{D}}_{1}\hat{P}\left(\zeta \right). \end{array}$$

## 3. Results and Discussion

**Figure 7:**
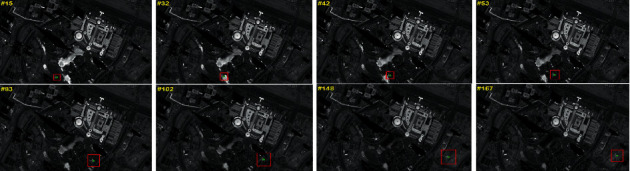
The results of moving target tracking by ISTC.

In this section, in order to verify the performance of the proposed method, the numerical simulations are conducted for the video satellite, where Jilin-1 is selected. The experiments are implemented in Matlab R2018b and NVIDIA GeForce GXT 2080Ti GPU. Firstly, the ISTC algorithm is employed to extract the image information. As shown in Figure 7, the results of moving target tracking by ISTC are presented. Meanwhile, based on this, the traditional sliding mode control and the HTFSMC are design comparative experiments. The initial condition of the simulations is demonstrated in Table 1. Besides, we can see that the image size is 4000 pixels × 4000 pixels, which is very large.

According to the above simulation parameters, we assume that the pixel coordinate system of the target located at (0, 510) in the initial time. Meanwhile, the traditional sliding mode control and the proposed controller are applied to obtain the variation curve of output torque, attitude angle, and angular velocity.

**Figure 8:**
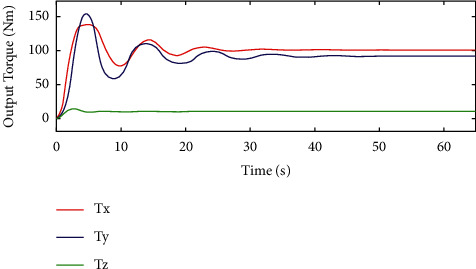
Variation curve of output torque by the traditional sliding mode controller.

**Figure 9:**
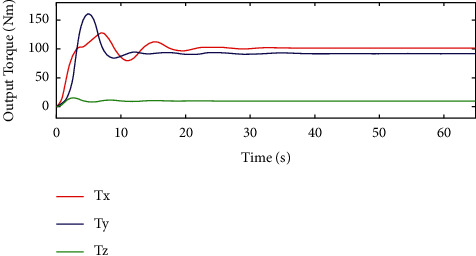
Variation curve of output torque by the HTFSMC.

In Figure 8, we can see that *T*_*x*_ and *T*_*y*_ are used to represent the output torque in the *x* and *y* directions and can converge after about 40s based on the sliding mode controller. However, in Figure 9, *T*_*x*_ and *T*_*y*_ can converge after 25s based on the HTFSMC. Compared with the sliding mode control, the proposed controller can improve the convergence speed. Besides, *T*_*z*_ is used to represent the output torque in the *z* direction and can converge faster after about 5s for these two controllers.

**Figure 10:**
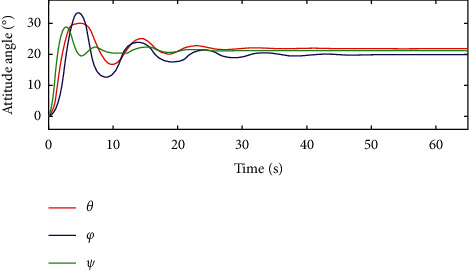
Variation curve of attitude angle by the traditional sliding mode controller.

**Figure 11:**
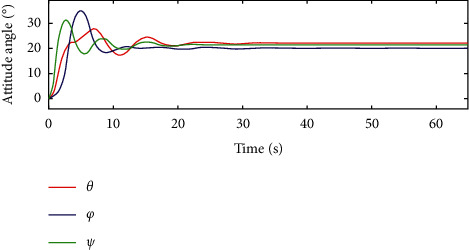
Variation curve of attitude angle by the HTFSMC.

In Figure 10 and Figure 11, for the traditional sliding mode controller and the HTFSMC, *θ* can converge after about 30s and 20s, respectively. *φ* can converge after about 40s and 20s, respectively. Ψ can converge after about 20s.

**Figure 12:**
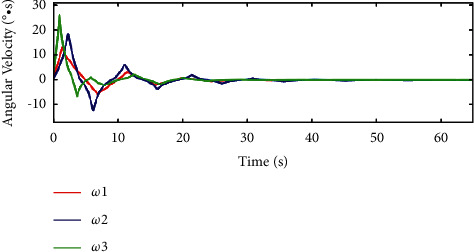
Variation curve of angular velocity by the traditional sliding mode controller.

**Figure 13:**
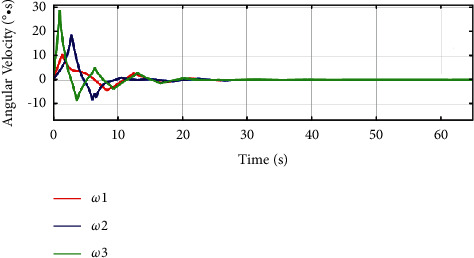
Variation curve of angular velocity by the HTFSMC.

In Figure 12 and Figure 13, for the traditional sliding mode controller and HTFSMC, *ω*_1_ can converge after about 30s and 23s, respectively. *ω*_2_ can converge after about 40s and 27s, respectively. *ω*_3_ can converge after about 30s and 23s, respectively.

**Figure 14:**
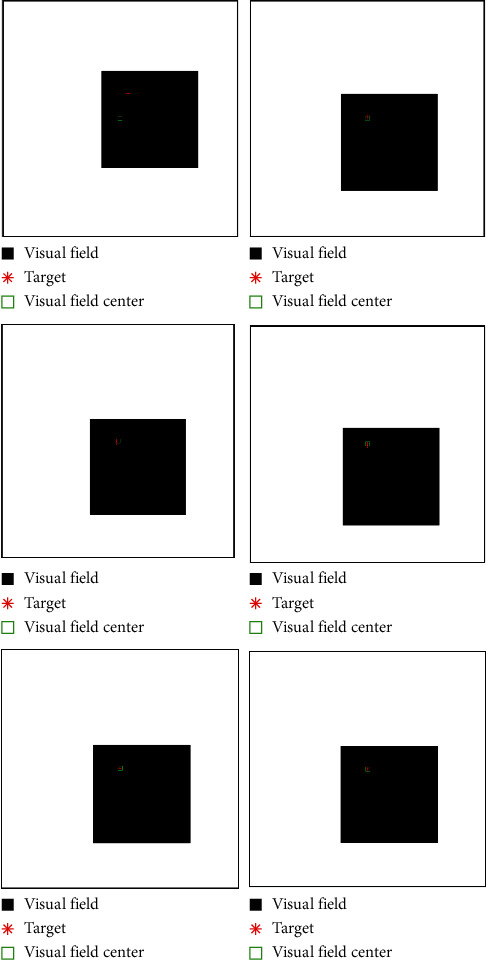
Simulation results of moving target gaze tracking based on the HTFSMC.

**Figure 15:**
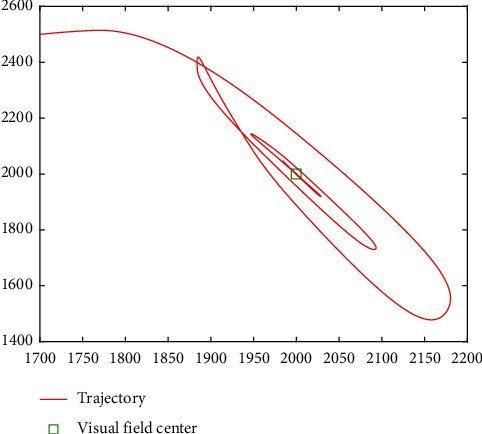
Trajectory of the target in image plane based on the HTFSMC.

**Figure 16:**
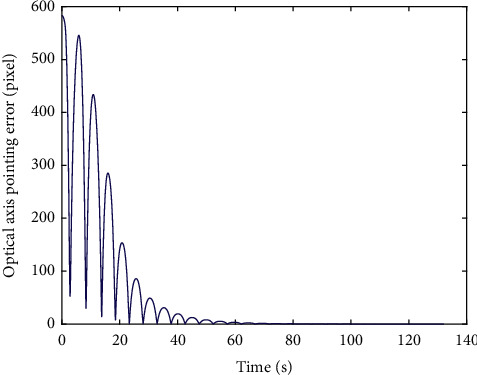
Optical axis pointing error based on the HTFSMC.

In Figure 14, the image information of the space target video is used as the input of the control loop. The image change caused by attitude adjustment is simulated. The image is scaled to 2000 pixels *∗* 2000 pixels, which represents the satellite visual field and shown in black. Besides, we design that the size of the actual image is 4000 pixels*∗* 4000 pixels. Meanwhile, the satellite visual field is embedded the actual image. The red *∗* is demonstrated the target and the green box visual field center. It is can be seen that the attitude control based on image information feedback can be achieved by the proposed controller. Moreover, the target imaging can be located in the center of the image plane to realize the gaze tracking control of the space target effectively. Accordingly, Figure 15 shows the trajectory of the target in image plane based on fuzzy sliding mode control. In addition, we can see that the position of the visual field center is (2000,2000). At first, the target position is not in the visual field center. The target imaging is located in the center based on the feedback control of image information.  Figure 16 shows the optical axis pointing error converging to 0 after about 60s based on the proposed controller.

**Figure 17:**
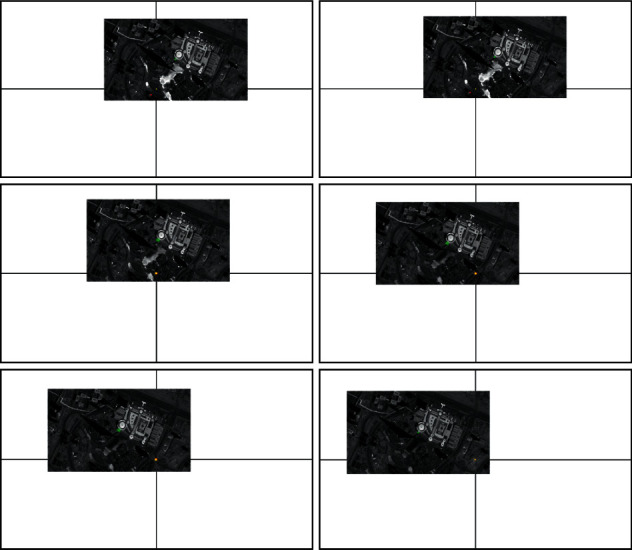
Simulation results of moving target gaze tracking based on the HTFSMC.

**Figure 18:**
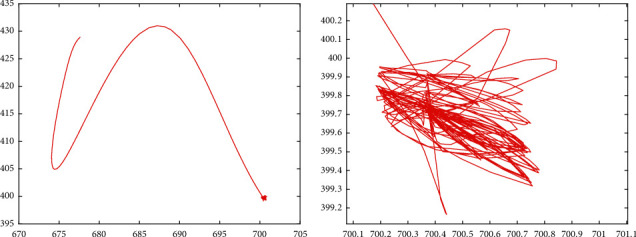
Trajectory of the target in image plane based on the HTFSMC: (a) trajectory of the target in the entire movement process; (b) local enlarged trajectory image of the target.

**Figure 19:**
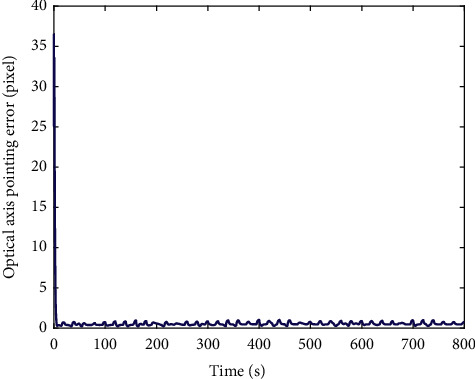
Optical axis pointing error based on the HTFSMC.

Figure 17 shows the simulation results of moving target gaze tracking in Jilin-1 video, in which the image changes caused by attitude adjustment are simulated and the moving target is the airplane. The moving airplane is not in the visual field center at the initial moment. The airplane is imaged in the visual field center based on the proposed controller of image information. Accordingly, Figure 18 shows the trajectory of the target in image plane based on fuzzy sliding mode control. Moreover, Figure 19 shows the optical axis pointing error converging to 0 quickly based on the proposed controller. It means that the gaze tracking of space moving target is effectively simulated.

## 4. Conclusions

The staring imaging attitude tracking and control for satellite videos based on image information is studied in this paper. An ISTC algorithm is designed to obtain the image information. Based on this, we introduced a HTFSMC law to achieve the attitude tracking and control. Furthermore, the hyperbolic tangent function and fuzzy logic system are introduced into the sliding mode controller.

In the experiments, the image information of the space target video sequence captured by Jilin-1 in orbit is used as the input of the control loop. The control part is realized through simulation. Compared with the traditional sliding mode controller, the image change caused by attitude adjustment is achieved successfully and quickly based on the proposed controller, and the target imaging can be located in the center of the image plane to realize the gaze tracking control of the space target effectively. In the future work, the space target video sequences will be used as the input of the control loop directly.

## Figures and Tables

**Table 1:** Initial conditions of the simulation.

Parameters	Parameter values
Inertia matrix	diag(264, 264, 28) kg *m*^*2*^
Initial attitude angle	[0°,0°,0°]
Initial attitude angle angular velocity	[0,0,0] rad/s
Pixel initial position	[1100,800]
Image size	4000 pixels × 4000 pixels
Disturbing torques	10^−6^N m
Image processing error	5 pixels
Pixel size	[8.38.3] mm
Camera focal length	4.2 mm

## Data Availability

The data used to support the findings of the study are available from the corresponding author upon request.
